# Case of Hydrocele Successfully Treated by Acupuncturation

**Published:** 1839-01-19

**Authors:** W. Harris


*Case of Hydrocele Successfully Treated by Acupuncturation.* By W. Harris, M. D.

A. B., aged sixteen years, in consequence of a slight injury, had an effusion of serum into his left tunica vaginalis testis, constituting hydrocele. His extreme modesty induced him to conceal his disease even from his own family, and to attempt to cure it himself. He procured a thumb lancet, opened the sac, and evacuated the fluid; the wound healed kindly, but at the end of three weeks, finding the collection of water greater in quantity than before, he repeated the same operation, and the same results followed. At the end of three more weeks, another experiment was tried. He concluded that if he could establish a permanent opening into the sac, the water would run out as fast as it collected, and a cure would eventually be accomplished. Accordingly he directed a *silversmith* to make him a silver tube, rather less than half an inch long, with a circular prominence at each end. He now opened the sac again with his lancet, allowed the water to escape, and then introduced his silver tube far enough to let the internal prominence rest against the internal surface of the tunica vaginalis testis, and to allow the external prominence to come in contact with the integuments. In this condition the surrounding parts healed close around the tube. After suffering great inconvenience from the perpetual fountain he had established, for several weeks, his clothes being constantly wet from the continued dribbling, and seeing no prospect of a cure, he came to my office, and consulted me on his case on the 5th of October last. After an examination of the part affected, I requested him to sit down, and to allow me, by dividing the integument on one side of the tube, to remove it. He replied that he would rather remove it himself, after he returned home. To this I consented, and directed him to apply adhesive plaster over the wound, and, after it was perfectly healed, to call again. As there was a good deal of irritation around the tube, and considerable thickening of the integument, I indulged a hope that the adhesive stage of inflammation was fairly set up within the vaginal tunic, and that a radical cure would follow the removal of the tube; but in this I was diappointed. When the patient called again, after a lapse of three weeks, the wound had healed, and there was a collection of about six ounces of water. Knowing that the attempts to affect a radical cure of this disease by *incision*, by the *seton*, and by *injections*, had often failed, and that *acupuncturation* had been followed by great success in the practice of Drs. Morand, Lewis, and others, I determined to try it in this case. And accordingly, I placed the patient in a sitting position, and directed him to seize the tumour by its posterior surface, and press the fluid forward, whilst I introduced an acupuncturation needle in eight different places upon the anterior and inferior surface of the tumour. Sometimes, upon withdrawing the needle, a very fine stream would spout forth for a few seconds, and again only a few drops. There was not, by this operation, more than half an ounce of fluid withdrawn, but the next day when the patient called, I found that the serum was extensively diffused through the cellular membrane between the integument and the tunic. At the end of a fortnight, the fluid in the cellular structure having been all absorbed, and there still remaining three or four ounces within the tunica vaginalis, I performed the same operation again, and with the same favourable results. This operation was repeated six times afterwards, at intervals of one week, with so much success, that the last time I saw my patient I did not think that there remained more than a drachm of fluid, and that another operation was unnecessary. I directed him to rub the scrotum with Linimentum Saponis twice a day, to keep it drawn up tight by a scrotal suspensory truss, with a view to promote the absorption of the little fluid that remained.

I directed the youth to call again, if he had any return of the disease, but as it is six weeks since, and he has not favoured me with another visit, I conclude, with confidence, that he is well.

